# Assessing Psychological Impact of COVID-19 among Parents of Children Returning to K-12 Schools: A U.S. Based Cross-Sectional Survey

**DOI:** 10.3390/healthcare10050775

**Published:** 2022-04-22

**Authors:** Kavita Batra, Jennifer R. Pharr, Emylia Terry, Brian Labus

**Affiliations:** 1 Department of Medical Education/Trauma and Critical Care, Kirk Kerkorian School of Medicine, University of Nevada, Las Vegas, NV 89102, USA; 2 Department of Environmental and Occupational Health, School of Public Health, University of Nevada, Las Vegas, NV 89119, USA; jennifer.pharr@unlv.edu (J.R.P.); emylia.terry@unlv.edu (E.T.); 3 Department of Epidemiology and Biostatistics, School of Public Health, University of Nevada, Las Vegas, NV 89119, USA; brian.labus@unlv.edu

**Keywords:** COVID-19, K-12, coronavirus anxiety, coronavirus obsession, psychosocial impact

## Abstract

Background and Purpose: While impacts of the pandemic on family well-being have been documented in the literature, little is known about the psychological challenges faced by children and their parents as schools reopen after mandated closures. Therefore, the purpose of this study was to determine if sending children back to in-person school impacts the mental health of parents and the perceived mental health of their children. Methods: This cross-sectional descriptive study recruited a nationally representative, non-probability sample of parents or guardians (*n* = 2100) of children attending grades K-12 in the United States (U.S.) through a 58-item web-based survey. The univariate, bivariate, and multivariate statistical tests were used to analyze the data. Results: The mean scores of parental Coronavirus anxiety and Coronavirus obsession were significantly different between race/ethnic groups of parents. Parents with children going to private schools had significantly higher mean scores for Coronavirus anxiety and obsession compared to parents whose children are attending public schools. Nearly 55% of parental Coronavirus anxiety was explained by the generalized anxiety, separation anxiety, child’s vulnerability to infection, and school type of the child. Similarly, 52% of parental Coronavirus obsession was explained by the generalized anxiety, separation anxiety, child’s vulnerability to infection, and social phobia of the children. Conclusions: The COVID-19 pandemic has a substantial impact on psychological well-being of parents and their school-going children. Findings of this study will inform policy makers in developing targeted interventions to address unique needs of families with school-going children.

## 1. Introduction

COVID-19 has negatively impacted mental health outcomes around the world. The pandemic has been associated with an increase in mental health disorders, elevated anxiety, and an overall disruption to the delivery of mental health services in most countries [1,2,3]. According to UNICEF (2021), one in five of individuals ages 15–24 reported feeling depressed and experiencing anhedonia (the inability to feel pleasure) during the pandemic [4]. Within the United States (U.S.), an increase in mental health disorders and symptoms have been attributed to COVID-19; communities of color, women, immigrants, and parents with school going children present higher rates of psychological symptoms [5,6,7].

While American adults were eight times more likely to suffer severe mental distress during the pandemic, adult caregivers and households with children reported even worse outcomes, including increased anxiety and depression-related symptoms, higher rates of substance use, and elevated suicidal ideation [5,8,9]. Almost half of parents reported higher levels of stress during the pandemic, and this rate increased to over 60% for parents with children attending remote learning environments [10]. In addition, nearly 50% of mothers whose children attended remote learning environments reported a decline in mental health, compared to 30% of fathers. Parents were more likely to access mental health services during the pandemic than non-parents with 75% of parents indicating a need for increased emotional support [10]. Parents were also more likely than non-parents to have been diagnosed with a mental health disorder and exhibited high rates of anxiety, depression, and burnout during the pandemic [10,11]. Job loss, health issues, and death of loved ones were attributed to lower levels of pandemic-related resilience and exacerbated mental health issues among parents [12]. The previous research highlighted a need for emotional, financial, and caregiving support to ease burdens on parents [11,12,13]. 

While 54% of parents indicated that their children could benefit from mental health services, considerably less is known about the needs and wellbeing of school going children during COVID-19 [10]. However, studies do show that COVID-19 reduced family functioning and children’s psychological wellbeing. He et al. found that caregiver stress during the pandemic was felt by many children, who internalized household discord and felt responsible for causing it [14]. Female students and older students have exhibited higher levels of anxiety during the pandemic [15]. In Canada, a quarter of children returning to in-person school had stress level above the critical threshold [16]. Further, nearly 25% of high school students in schools with closures reported elevated levels of pandemic-related worry, with students of color, low-income students, students in higher grades, and female students exhibiting the highest rates of COVID-19-related concerns [17]. As people of color and people with low income experienced higher rates of COVID-19, the elevated levels of pandemic-related worry among students of color and low-income students are additional pandemic-related disparities [17]. 

While pandemic impacts on family wellbeing have been documented in the literature [10,11,12,13,14,15], little is known about the mental health challenges faced by children and their parents as schools across the country reopen after mandated closures. Therefore, the purpose of this study was to determine if sending children back to in-person school impacts the mental health of parents and the perceived mental health of their children as they return to school. Due to the established relationship between child–parent anxiety and phobias, we wanted to understand this relationship within the context of COVID-19 and children’s return to school [18]. Here, we explore the association between parental COVID-19 anxiety, obsession, and anxiety (generalized and separation) and social phobia in their children. As schools reopened across the country and children transitioned away from home schooling, we hypothesized that their generalized anxiety, separation anxiety, and social phobia would be associated with COVID-19 anxiety and obsession in their parents. 

## 2. Materials and Methods

### 2.1. Study Design and Eligibility Criteria

This cross-sectional descriptive study recruited a nationally representative sample of parents or guardians of children who were enrolled in grades K-12 (kindergarten, elementary, middle, high school) in the U.S. In addition, only participants who could comprehend English and were capable of providing voluntary consent were included in this study.

### 2.2. Data Collection and Sampling Procedure

Data were collected between November and December 2021. This study utilized a commercial service offered through the Qualtrics Research Marketing Team to manage the data collection. A contractual agreement was established between the study’s investigators and Qualtrics to recruit a quota sample that would mirror the U.S. Census with regard to gender, race, and region of the country of participating parents. Qualtrics recruited a high-quality sample through multiple avenues, including apps, games, social media platforms, and their dashboard-type system. A detailed sampling strategy used by the Qualtrics can be found at https://www.qualtrics.com/panels-project/ (accessed on 18 January 2022) [19]. A few screening questions related to the study’s inclusion criteria were asked at the start of the survey to determine eligibility of the participants and to prevent response bias. If participants did not self-identify as a parent or guardian of a child going to school in grades K-12, the survey was programmed to automatically terminate. Given the use of multiple sources for the data collection, calculation of the response rate was not possible. Eligible participants who completed the survey were given incentives per terms and conditions set forth by Qualtrics and its data collection partners.

### 2.3. Ethical Considerations

This study (protocol ID: UNLV-2021-223 dated 2 December 2021) was granted an exempt status from the Institutional Review Board at the University of Nevada, Las Vegas. This study was exempted under category 2, as it includes protocols survey procedures, interviews, observation of public behavior, etc. Participants were provided detailed information about the study objectives, and participation was voluntary. Personal identifiers of the participants were not collected to abide by the ethical guidelines.

### 2.4. Quality Assurance of Data

The Qualtrics Research Marketing Team provided a complete dataset with only complete survey responses as a part of the contractual agreement. Qualtrics first collected only 10 percent of the total sample size as a part of a “soft launch” to identify any potential discrepancies before the full data set was collected [19]. Once the data quality was assessed by the study’s investigators, the rest (90%) of the sample was collected. Algorithms such as digital fingerprinting and “prevent ballot-box stuffing” were used to ensure unique responses (only one response per participant). Once the data collection effort was completed, investigators were given an additional 7 days to review the quality of the data. 

### 2.5. Survey Instrument

The 58-item questionnaire included 5 items related to parental Coronavirus anxiety, (Appendix A.1), 4 items related to parental Coronavirus obsession (Appendix A.2), and 22 items to measure separate anxiety, social phobia, and generalized anxiety among children using the Revised Child Anxiety Disorder Scale-Parent version (RCADS-P) [20,21,22,23]. As described by the previous studies, Coronavirus anxiety is an unhealthy state of mind, which stems from behavioral and psychological reactions following exacerbation of fear and worries related to coronavirus and its stimuli [24]. Coronavirus, on the other hand, is repetitive and maladaptive thinking about COVID-19 most of the time (Lee, 2020a; Lee 2020b; Chen et al., 2021). The remaining items were related to school safety measures and demographics of parents and their youngest child. Both Coronavirus anxiety and obsession were measured on a 5-point Likert scale ranging from “not at all” (0) to “nearly every day over the last 2 weeks” (4) [20,21]. Separation anxiety, social phobia, and generalized anxiety were measured on a 4-point Likert scale ranging from “never” (0), “sometimes” (1), “often” (2), and “always” (3) [19]. The RCADS-P has a good internal consistency, test–retest reliability, and construct validity [22,23].

### 2.6. Sample Size Justification

Prior to running analyses, the minimum sample size required was predetermined using the formula *n* = (z)2 *p* (1 − *p*)/d^2^ with a 95% confidence interval (alpha = 0.05, Z = 1.96), and a margin of error d = 5% [25]. This sample size calculation used the normal approximation to the binomial distribution [21]. The proportion (*p*) of worst mental health symptoms among parents of school going children was 27% in June 2020 based on the data reported by a study performed by Patrick et al. [26]. The estimated sample size after accounting for 10% non-response was 334. We also predetermined our sample size separately for t, chi-square, ANOVA, and multiple regression tests by using “small” Cohen’s effect size conventions (effect size = 0.1 for *t*-tests, chi square, one-way ANOVA; 0.02 for linear regression with 10 predictors) [27]. The total sample size estimated with a power of 0.95 for was 1289, 1979, 1865, and 1229 for t test, chi square, one-way ANOVA, and linear regression test, respectively. The sample size with the greatest value (*n* = 1.979) was considered appropriate since it satisfies the minimum requirement of all the statistical tests used.

### 2.7. Data Analysis

**Figure 1:**
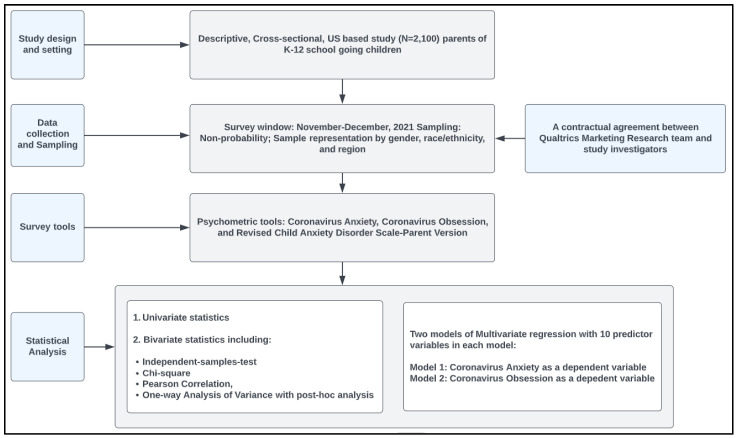
Flowchart detailing the study methodology.

SPSS software v.26 (IBM Corp.: Armonk, NY, USA) was used to analyze the data. Univariate statistics were used to describe the sample population. Independent-samples-t-test/Welch test or one-way ANOVA was used to draw group comparisons for continuous outcomes. Homogeneity of variance assumption was assessed by the Levene’s test. Tukey or Games–Howell analysis was conducted to derive multiple group comparisons (wherever appropriate). The Pearson test was conducted to assess bivariate associations. Two models of multiple linear regression were fit to predict parental Coronavirus anxiety and obsession by parent demography, child demography, school characteristics, and the child’s generalized anxiety, social phobia, and separation anxiety. All independent variables, including the polytomous variables, were dummy coded to aid in the accurate computations of regression coefficients and slopes. A Checklist for Statistical Assessment of Medical Papers (CHAMP statement) was used for our reporting [28]. The significance level was set at 5%. A detailed methodology can be seen in Figure 1.

## 3. Results

### 3.1. Univariate Statistics

A total of 2100 parents completed the survey. The mean age of the participants was 49.9 ± 11.2 years. The gender, race/ethnicity, and regional distribution was comparable to the U.S. Census parameters. Over thirty percent of parents had a 4-year college degree followed by nearly 27% reporting they had attended some college (Table 1). Over sixty percent of parents were married and had full-time paid jobs (Table 1). As reported by the parents, nearly 51% of children were male as compared to 47% being female (Table 2). The mean age of the youngest child reported was 9.10 ± 4.11 years and over 40% of children were attending elementary school (Table 2). A larger proportion (83.4%) of parents reported having their children attending public schools. Over 35% of parents reported having a child vulnerable to infections or were living with a vulnerable family member. Nearly 57% of parents relied on local health departments for COVID-19 related information. 

### 3.2. Bivariate Statistics

A Welch-t-test was run to determine if there were differences in mean scores of Coronavirus anxiety, Coronavirus obsession, children separation anxiety, and generalized anxiety by parental gender. There were no outliers in the data, as assessed by inspection of a boxplot. All scores for each level of gender were normally distributed, as assessed by Shapiro–Wilk’s test (*p* > 0.05), and there was no homogeneity of variances as assessed by Levene’s test for equality of variances (*p* < 0.05). For social phobia, the assumption of homogeneity of variance was not met (*p* > 0.564), for which an independent-samples-t-test was used to compare the means. The differences in the means scores of parental Coronavirus anxiety and Coronavirus obsession, and children separation anxiety, social phobia, and generalized anxiety by parental gender, were not significant (Table 3). A one-way Welch ANOVA was conducted to compare mean scores among different race/ethnic groups of parents. There were no outliers, and the data were normally distributed for each group, as assessed by boxplot and Shapiro–Wilk test (*p* > 0.05), respectively. Homogeneity of variances was violated, as assessed by Levene’s Test of Homogeneity of Variance (*p* < 0.001). Coronavirus anxiety, Welch’ F (3, 2096) = 14.829, *p* < 0.001, ω2 = 0.02, and Coronavirus obsession, F (3, 2096) = 8.536, *p* < 0.001, ω2 = 0.011, scores were significantly different between race/ethnic groups of parents. Coronavirus anxiety score increased from the other group (M = 3.30, SD = 4.37) to the Black (M = 4.20, SD = 4.96), White (M = 4.60, SD = 5.24), and Hispanic (M = 6.00, SD = 5.32) groups, in that order. Games-Howell post hoc analysis revealed that the mean score increases from Black to Hispanic (1.77, 95% CI [0.72, 2.82]), White to Hispanic (1.36, 95% CI [0.55, 2.17]), as well as White to other (1.30, 95% CI [0.49, 2.11]) were significant. Similarly, the mean scores of separation anxiety, social phobia, and generalized anxiety of children (reported by parents) were significantly different, with Hispanic parents reporting the highest scores (Table 3). The assumption of homogeneity of variance was met for groups of parents living in different regions (*p* = 0.06). By regional location of parents, only Coronavirus obsession mean scores were significantly different. F (3, 2096) = 3.455, *p* = 0.02, ω2 = 0.003. Games-Howell post hoc analysis revealed that the mean increases from Midwest to Northeast region (0.93, 95% CI [0.14, 1.72]), as well as Midwest to Southern region (0.67, 95% CI [0.017, 1.33]) were significant (*p* < 0.05). There was no homogeneity of variances in, as assessed by Levene’s test for equality of variances (*p* < 0.05) by school type. Parents with children going to private schools had significantly higher mean scores for Coronavirus anxiety and obsession compared with parents whose children are attending public schools (Table 3). In addition, parents of children going to private schools reported that their children had higher separation anxiety, social phobia, and generalized anxiety as compared to those going to public schools (Table 3). Children’s separation anxiety (as reported by the parents), F (3, 2096) = 15 139, *p* < 0.001, ω2 = 0.020, scores were significantly different by school level of the children. Separation anxiety score decreased from the Kindergarten (M = 7.05, SD = 5.19) to the Elementary (M = 6.17, SD = 5.00), Middle (M = 5.81, SD = 5.78), and High (M = 4.67, SD = 5.29) school level, in that order. The mean scores of all the constructs were significantly different by the gender of the child with parents having male children and other gender identities reporting higher scores for coronavirus anxiety and obsession (Table 3).

Table 4 shows the Pearson correlation coefficient matrix of all continuous variables. Parental Coronavirus anxiety was directly and strongly correlated with Coronavirus obsession (r = 0.83, *p* < 0.01), separation anxiety of the child (r = 0.71, *p* < 0.01), and had direct moderate correlation with social phobia (r = 0.62, *p* < 0.01), and generalized anxiety of the child (r = 0.66, *p* < 0.01). Parental age was negatively or indirectly correlated with parental anxiety and obsession as well as separation anxiety, social phobia, and generalized anxiety of the child. 

### 3.3. Multiple Linear Regression

For two models with parental Coronavirus anxiety and Coronavirus obsession as dependent variables, there was independence of residuals, as assessed by a Durbin–Watson statistic nearly 2. There was homoscedasticity, as assessed by visual inspection of a plot of studentized residuals versus unstandardized predicted values. There was no evidence of multicollinearity, as assessed by tolerance values greater than 0.1. There were no studentized deleted residuals greater than ±3 standard deviations, no leverage values greater than 0.2, and values for Cook’s distance above 1. The assumption of normality was met, as assessed by a P-P Plot (Appendix B.1. and Appendix B.2). The multiple regression model was significant for parental coronavirus anxiety, F (15, 2048) = 169.051, *p* < 0.001, adj. R^2^ = 0.55. Only generalized anxiety of the child, separation anxiety of the child, child’s vulnerability to infection, and school type variables added significantly to the prediction, *p* < 0.05. Regression coefficients and standard errors can be found in Table 5. The multiple regression model was significant for parental coronavirus obsession, F (15, 2048) = 151.182, *p* < 0.001, adj. R^2^ = 0.522. Only generalized anxiety, separation anxiety, child’s vulnerability to infection, and social phobia variables added significantly to the prediction, *p* < 0.05. Regression coefficients and standard errors can be found in Table 6. 

## 4. Discussion

This study yielded several interesting findings. First, there was a variation in parental COVID-19 anxiety and obsession as well as child separation anxiety, social phobia, and generalized anxiety based on the parent’s race/ethnicity, with Hispanic parents reporting the highest scores for all variables. Hispanic and Latino populations in the U.S. have been disproportionately impacted by COVID-19 [29]. Compared with White people, Hispanic/Latino people have had 1.5 times more cases, 2.4 times more hospitalizations, and 1.9 times more deaths [25]. These disparities in COVID-19 cases, hospitalizations, and deaths may have influenced Hispanic/Latino parents’ COVID-19 anxiety and obsession and their children’s anxiety and social phobia. Another study found that Hispanic parents were less likely to support schools reopening full-time/in person and were more likely to support homeschooling until a vaccine was available and mask mandates in school than White parents [29,30]. Additionally, one study of Latino parenting styles revealed that the majority of parents had a protective/warm parenting style, while another found that Latino parents are more likely to have a family focused parenting style with high scores for involvement, monitoring, agency, and familismo (the cultural orientation and sense of obligation to family) [31,32,33,34]. These might explain higher scores for COVID-19 anxiety and obsession among this parent group compared with the other groups. 

COVID-19 obsession among parents varied by region, with parents living in the Northeast having the highest scores and parents living in the Midwest having the lowest scores. This may have been impacted by the rates of disease or the political response such as mask or vaccine mandate variations within the regions. The Northeast, which includes New York, was hit hard by the pandemic in the early months. This may have influenced people’s attitudes about the seriousness of COVID-19 in the Northeast region [35]. Recent hotspot trends in COVID-19 cases show relatively low cases in the Midwest region of the country compared with other regions [35]. This may be a result of population density, as many of the Midwest states have large rural areas. People in the Midwest might not feel as threatened by COVID-19 as people living in the more densely packed Northeast part of the country. Interestingly, the Northeast was reported to have the highest rate of mask wearing than other regions in the U.S. in June of 2020 [36]. Additionally, states that comprise the Northeast regions have the highest rates of vaccination according to the Johns Hopkins COVID Resource Center [37]. This higher COVID-19 vigilance may have led to more persistent thoughts about COVID-19 among people in the Northeast. Lastly, the survey took a few weeks to conduct, and COVID rates were in a constant state of flux in November and December, 2021. These differences could have affected responses. For example, news coverage of a pediatric death in the community could have driven people to be more worried that day

Parents whose children attended private school had higher scores for all constructs than parents whose children attended public schools. A large survey of school attendance in the U.S. during November–December of 2020 found that children attending private school were much more likely to attend school fully in-person and face to face (60%) compared with students in public school with virtual attendance (25%) [38]. Another study found that attending a private school is associated with almost a 40% increase in attending school fully in-person and almost a 30% decrease in attending school fully remotely when compared with public school attendance. These differences in full in-person attendance may have heightened parents’ anxiety and obsession with COVID-19 as well have been associated with children’s anxiety and social phobia. On the other hand, some parents who were anxious about COVID-19 and how prevention strategies were being handled in the public-school system sought out private school for their children [38]. Additionally, parents of children who have social phobia or generalized anxiety may be more likely to send their children to private school because of smaller class sizes and more resources for children with special needs, which may explain some of the difference in the reported children’s anxiety and social phobia scores [39]. 

Parents’ COVID-19 anxiety was significantly associated with their child’s reported generalized anxiety and separation anxiety while parents’ COVID-19 obsession was significantly associated with their child’s reported generalized anxiety, separation anxiety, and social phobia. Due to the cross-sectional nature of this study, we cannot say if parents’ COVID-19 anxiety and obsession resulted in higher anxieties and social phobia among children or if children’s higher anxieties and social phobia created higher COVID-19 anxiety and obsession among parents. It could be either/or, or both. During the COVID-19 pandemic, parents have been more likely to have higher rates of anxiety and depression than non-parents [10,11]. Research has shown that parental anxiety increases the risk of anxiety and other mood disorders among their children [40,41]. Parental stress has been associated with distress in their children during the COVID-19 pandemic [14]. Additionally, when their child experiences distress, anxious parents tend to reciprocate their child’s distress rather than help them regulate it [42]. Among parents who reported having high reactivity to imagining their child experiencing fear, greater parental anxiety was associated with higher reactivity in their children. During the distress associated with COVID-19, parents’ and children’s reactivity to the distress may have elevated anxiety in each [42].

### Strengths and Limitations

As with any study, there are limitations to this study. Due to the cross-sectional nature of this study, causation could not be assessed. Additionally, we did not measure pre-COVID-19 measures of anxiety in parents or children and are not able to determine if anxiety increased during the pandemic. Parents who are more anxious may have been more likely to complete the survey which may have resulted in anxiety bias. Parents self-reported their COVID-19 anxiety and obsession which subjects this study to a self-report bias. Parents may have over or under reported their concerns. Additionally, we did not directly measure children’s general and separation anxiety or social phobia, but rather had parents reported these data. Parents may have over or under reported their child’s anxieties and social phobia based on their own perceptions or concerns. Despite these limitations, there are strengths to this study. We had a nationally representative sample of parents mirroring the census representation by gender, race/ethnicity, and region distribution, although generalizability may be somewhat compromised by the lack of representation by type of child’s school. Still, the findings of this study provide baseline data to design targeted interventions.

## 5. Conclusions

The purpose of this study was to determine if sending children back to in-person school impacts the mental health of parents and the perceived mental health of their children. The mean scores of parental Coronavirus anxiety and Coronavirus obsession were significantly different between race/ethnic groups of parents and among parents with children going to private versus public schools. Over half of parental Coronavirus anxiety was explained by the generalized anxiety, separation anxiety, and school type of the child, while parental Coronavirus obsession was explained by the generalized anxiety, separation anxiety, and social phobia of the children. The COVID-19 pandemic has a substantial impact on psychological well-being of parents and their school-going children. Future studies could directly measure children’s anxiety, separation anxiety, and social phobia rather than gleaning this data from parents. A future area for research may include children prior to returning to school, during the return, and after the return to school to draw comparisons. Additionally, research in this area should continue as the COVID-19 pandemic and precautions pertaining to the pandemic change over time. Findings of this study can inform policy makers to develop targeted interventions to address unique needs of families with school-going children. For example, parents of children in private school who were more likely to be in-person, face to face, may need more information about the precautions being implemented to keep their children safe while in school (e.g., social distancing, mask requirements, protective barriers). This information needs to be culturally tailored toward different races and ethnicities with the understanding that Hispanic/Latino parents may have elevated levels of concern. Due to the association between parental Coronavirus anxiety/obsession and children’s anxiety (general and separation) and social phobia, interventions to increase parents’ coping skills (e.g., mindfulness, mediation) or stress reduction (emotional, financial, or caregiving support) may help to reduce anxiety for both parents and children.

## Figures and Tables

**Table 1:** Demographic characteristics of the respondents (*n* = 2100).

Variable	Categories	*n* (%)	95% CI (LCL, UCL)
Age (Mean ± SD)	-	49.9 ± 11.2	49.4, 50.4
Gender	Female	1065 (50.7)	48.5, 52.9
	Male	999 (47.6)	45.4, 49.7
	Other, including non-binary, Transgender	29 (1.4)	0.93, 2.0
Race/ethnicity	Non-Hispanic White	1197 (57.0)	54.9, 59.1
	Non-Hispanic Black	273 (13.0)	11.6, 14.5
	Hispanic	378 (18.0)	16.4, 19.7
	Asian or Pacific Islanders	126 (6.0)	5.0, 7.1
	Other	126 (6.0)	5.0, 7.1
Education	Some high school	54 (2.6)	1.9, 3.3
	High school diploma or GED	417 (19.9)	18.2, 21.6
	Some College	560 (26.7)	24.8, 28.6
	4-year college degree	656 (31.2)	29.3, 33.3
	Graduate level degree	383 (18.2)	16.6, 19.9
	Other	30 (1.4)	1.0, 2.0
Marital status	Married	1294 (61.6)	59.5, 63.7
	Single, never married	364 (17.3)	15.7, 19.0
	Divorced or Separated	192 (9.1)	7.9, 10.5
	Widowed	42 (2.0)	1.4, 2.7
	A member of an unmarried couple	208 (9.9)	8.6, 11.3
Employment status	Full-time paid job	1390 (66.2)	64.1, 68.2
	Part-time paid job	175 (8.3)	7.2, 9.6
	Self-employed	146 (7.0)	5.9, 8.1
	Unable to work	103 (4.9)	4.0, 5.9
	Unemployed and looking for work	104 (5.0)	4.1, 5.9
	Unemployed but not looking for work (e.g., retired, homemaker, student, etc.)	182 (8.7)	7.5, 10.0
Annual Gross Income	0 to $10,000	153 (7.3)	6.2, 8.5
	$10,001 to $25,000	202 (9.6)	8.4, 11.0
	$25,001 to $50,000	473 (22.5)	20.8, 24.4
	$50,001 to $100,000	606 (28.9)	26.9, 30.9
	$100,001 to $250,000	528 (25.1)	23.3, 27.1
	>$250,001	111 (5.3)	4.4, 6.3
Region	Midwest	448 (21.3)	19.6, 23.2
	Northeast	358 (17.0)	15.5, 18.7
	South	819 (39.0)	36.9, 41.1
	West	475 (22.6)	20.8, 24.5

SD: Standard Deviation; CI: Confidence interval; LCL: Lower Confidence Level; UCL: Upper Confidence Level. The percentages may not add up to 100% for some variables (e.g., gender and income), as a few participants preferred not to say.

**Table 2:** Characteristics of family and youngest school going child as reported by parent respondents (*n* = 2100).

Variable	Categories	*n* (%)	95% CI (LCL, UCL)
Age of the youngest child (Mean ± SD)	-	9.10 ± 4.11	8.93, 9.27
Gender of the youngest child	Female	983 (46.8)	44.7, 49.0
	Male	1077 (51.3)	49.1, 53.4
	Other, including non-binary, transgender	26 (1.2)	0.8, 1.8
School type of the youngest child	Private	349 (16.6)	15.1, 18.3
	Public	1751 (83.4)	81.7, 85.0
School level of the youngest child	Kindergarten	536 (25.5)	23.7, 27.5
	Elementary	852 (40.6)	38.5, 42.7
	Middle school	357 (17.0)	15.4, 18.7
	High school	355 (16.9)	15.3, 18.6
Vulnerability of the youngest child to infections	Yes	741 (35.3)	33.2, 37.4
	No	1094 (52.1)	50.0, 54.3
	Not sure	265 (12.6)	11.2, 14.1
Living with vulnerable family member	Yes	755 (36.0)	33.9, 38.1
	No	1345 (64.0)	62.0, 66.1
Friends/family tested positive for COVID-19	Yes	955 (45.5)	43.3, 47.6
	No	1129 (53.8)	51.6, 55.9
COVID-19 information source	Local health department	1194 (56.9)	54.7, 58.9
	School administration	367 (17.5)	15.9, 19.2
	Parent association	205 (9.8)	8.5, 11.1
	Superintendent or official district communications	166 (7.9)	6.8, 9.1

The percentages may not add up to 100% for some variables (e.g., gender and family member tested positive for COVID-19), as a few participants preferred not to say. CI: Confidence interval; LCL: Lower Confidence Level; UCL: Upper Confidence Level.

**Table 3:** Group-wise comparisons for parental and children anxiety as reported by parents (*n* = 2100).

Variable	Groups	Coronavirus Anxiety		Coronavirus Obsession		Separation Anxiety		Social Phobia		Generalized Anxiety	
		M ± SD *p* Value		M ± SD *p* Value		M ± SD *p* Value		M ± SD *p* Value		M ± SD *p* Value	
Gender of the parent	Male	4.64 ± 5.42	0.8	4.60 ± 4.66	0.3	6.21 ± 5.61	0.2	8.93 ± 6.68	0.6	5.22 ± 4.73	0.8
	Female	4.60 ± 4.93		4.39 ± 4.23		5.93 ± 4.98		9.07 ± 6.55		5.19 ± 4.43	
Race/ethnicity of the parent	White	4.60 ± 5.24	**<0.001**	4.37 ± 4.48	**<0.001**	6.12 ± 5.36	**<0.001**	9.18 ± 6.46	**<0.001**	5.33 ± 4.54	**<0.001**
	Black	4.20 ± 4.96		4.38 ± 4.39		5.62 ± 5.26		8.34 ± 7.03		4.45 ± 4.31	
	Hispanic	6.00 ± 5.32		5.51 ± 4.51		7.07 ± 5.31		10.09 ± 6.89		6.27 ± 4.87	
	Other	3.30 ± 4.37		3.89 ± 4.01		4.91 ± 4.68		7.46 ± 6.07		4.06 ± 4.26	
Region	Northeast	5.05 ± 5.30	0.05 *	4.93 ± 4.43	**0.02**	6.37 ± 5.62	0.08	9.37 ± 7.08	0.3	5.53 ± 4.94	0.09
	South	4.74 ± 5.15		4.67 ± 4.52		6.12 ± 5.16		8.89 ± 6.48		5.13 ± 4.48	
	West	4.66 ± 5.29		4.44 ± 4.48		6.31 ± 5.50		9.33 ± 6.90		5.53 ± 4.84	
	Midwest	4.07 ± 4.94		3.99 ± 4.24		5.54 ± 4.99		8.66 ± 6.11		4.88 ± 4.16	
School type	Public	4.40 ± 5.06	**<0.001**	4.38 ± 4.39	**0.002**	5.85 ± 5.19	**<0.001**	8.84 ± 6.50	**0.005**	5.10 ± 4.46	**0.006**
	Private	5.81 ± 5.53		5.21 ± 4.63		7.25 ± 5.64		9.98 ± 7.04		5.91 ± 5.12	
School level	Kindergarten	5.05 ± 5.25	0.2	4.75 ± 4.45	0.4	7.05 ± 5.19	**<0.001**	8.48 ± 6.66	0.09	4.90 ± 4.59	0.2
	Elementary	4.53 ± 5.00		4.47 ± 4.27		6.17 ± 5.00		9.14 ± 6.25		5.28 ± 4.33	
	Middle school	4.63 ± 5.30		4.55 ± 4.61		5.81 ± 5.78		9.59 ± 6.97		5.56 ± 4.89	
	High school	4.25 ± 5.28		4.24 ± 4.57		4.67 ± 5.29		9.00 ± 6.95		5.30 ± 4.83	
Gender of the youngest child	Male	4.77 ± 5.20	**<0.001**	4.58 ± 4.50	**0.03**	6.27 ± 5.44	**0.04**	9.20 ± 6.65	**0.003**	5.45 ± 4.64	**<0.001**
	Female	4.37 ± 5.07		4.38 ± 4.33		5.82 ± 5.11		8.74 ± 6.55		4.91 ± 4.47	
	Other	8.35 ± 6.01		6.62 ± 4.64		7.73 ± 5.30		12.96 ± 5.62		9.23 ± 4.65	

Note: For two groups comparisons, independent-sample-*t*-test was conducted. For more than two groups, one-way ANOVA was conducted. Bolded *p* values are significant <0.05 level. * *p* < 0.05.

**Table 4:** Pearson correlations, and reliability estimates for study variables in the sample (*n* = 2100).

Variables	1	2	3	4	5	6
1. Coronavirus anxiety of parents	1	0.83 **	0.71 **	0.62 **	0.66 **	−0.14 **
2. Coronavirus obsession of parents	0.83 **	1	0.70 **	0.62 **	0.64 **	−0.13 **
3. Separation anxiety of child	0.71 **	0.70	1	0.75 **	0.76 **	−0.19 **
4. Social phobia of child	0.62 **	0.62 **	0.75 **	1	0.82 **	−0.12 **
5. Generalized anxiety of child	0.66 **	0.64 **	0.76 **	0.82 **	1	−0.11 **
6. Parental age	−0.14 **	−0.14 **	−0.19 **	−0.12 **	−0.11 **	1
Cronbach’s Alpha	0.915	0.895	0.901	0.925	0.918	-

** *p* < 0.01.

**Table 5:** Multiple regression results for Coronavirus anxiety among parents.

Variables	B	95% CI for B		SE B	β	R^2^	ΔR^2^
		LL	UL				
Model	-					0.553	0.550 **
Constant	−0.020	−0.826	0.785	0.411	-		
Parental gender (ref: female)	−0.157	−0.494	0.179	0.172	−0.015		
Parental age	−0.007	−0.023	0.010	0.009	−0.012		
Race/ethnicity of the parent, white (ref: black)	0.016	−0.454	0.486	0.240	0.002		
Race/ethnicity of the parent, Hispanic (ref: black)	0.540	−0.016	1.096	0.283	0.040		
Race/ethnicity of the parent, other (ref: black)	−0.379	−0.985	0.226	0.309	−0.024		
Child gender, male (ref: female)	0.070	−0.257	0.397	0.167	0.007		
Child gender, other (ref: female)	1.870	−0.044	3.784	0.976	0.029		
Child’s vulnerability to infections (ref: No)	0.768	0.433	0.999	0.171	0.071 **		
School type (ref: public)	0.460	0.051	0.869	0.209	0.028 *		
School level, elementary (ref: kindergarten)	−0.128	−0.517	0.260	0.198	−0.012		
School level, middle (ref: kindergarten)	0.052	−0.436	0.541	0.249	0.004		
School level, high (ref: kindergarten)	0.312	−0.194	0.819	0.258	0.023		
Social phobia of child	0.042 *	0.002	−0.084	0.022	0.053		
Generalized anxiety of child	0.25 **	0.19	0.32	0.032	0.22 **		
Separation anxiety of the child	0.467 **	0.417	0.52	0.025	0.48 **		

Note. Model = “Enter” method in SPSS Statistics; B = Unstandardized regression coefficient; CI = confidence interval; LL = lower limit; UL = upper limit; SE B = Standard error of the coefficient; β = standardized coefficient; R^2^ = coefficient of determination; Δ R^2^ = adjusted R; * *p* < 0.05; ** *p* < 0.01.

**Table 6:** Multiple regression results for Coronavirus obsession among parents.

Variables	B	95% CI for B		SE B	β	R^2^	Δ R^2^
		LL	UL				
Model	-					0.525	0.522 **
Constant	0.731	0.018	1.444	0.364			
Parental gender (ref: female)	0.166	−0.132	0.464	0.152	0.019		
Parental age	−0.008	−0.023	0.007	0.008	−0.018		
Race/ethnicity of the parent, white (ref: black)	−0.325	−0.741	0.091	0.212	−0.036		
Race/ethnicity of the parent, Hispanic (ref: black)	0.139	−0.353	0.631	0.251	0.012		
Race/ethnicity of the parent, other (ref: black)	0.013	−0.523	0.549	0.273	0.001		
Child gender, male (ref: female)	−0.174	−0.464	0.116	0.148	−0.020		
Child gender, other (ref: female)	−0.145	−1.839	1.550	0.864	−0.003		
Child’s vulnerability to infections (Ref: No)	0.653 **	0.357	0.950	0.151	0.070 **		
School type (ref: public)	0.053	−0.310	0.415	0.185	0.004		
School level, elementary (ref: kindergarten)	0.009	−0.335	0.353	0.175	0.001		
School level, middle (ref: kindergarten)	0.126	−0.306	0.559	0.221	0.011		
School level, high (ref: kindergarten)	0.357	−0.092	0.805	0.229	0.030		
Social phobia of child	0.086 **	0.048	0.123	0.019	0.128 **		
Generalized anxiety of child	0.176 **	0.120	0.232	0.029	0.181 **		
Separation anxiety of the child	0.369 **	0.326	0.413	0.022	0.440 **		

Note. Model = “Enter” method in SPSS Statistics; B = Unstandardized regression coefficient; CI = confidence interval; LL = lower limit; UL = upper limit; SE B = Standard error of the coefficient; β = standardized coefficient; R^2^ = coefficient of determination; ΔR^2^ = adjusted R; ** *p* < 0.01.

**Figure A1:**
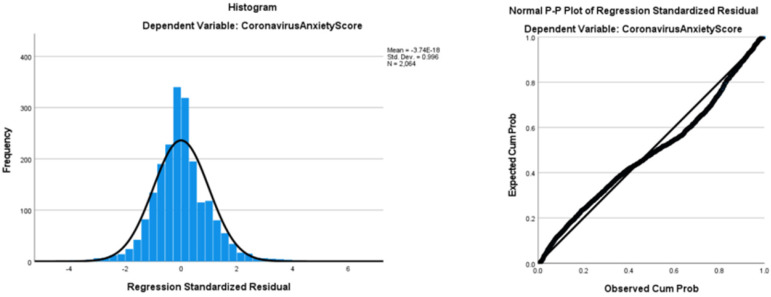
Figures showing residual analysis with Coronavirus Anxiety as dependent variable.

**Figure A2:**
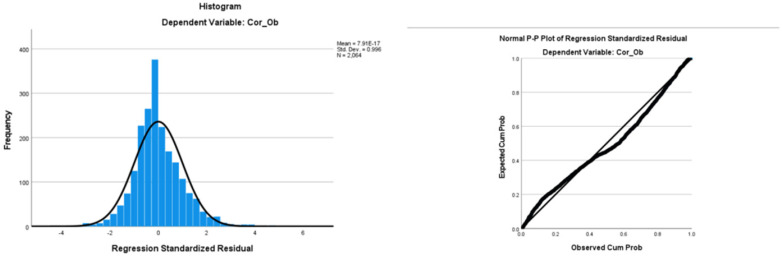
Figures showing residual analysis with Coronavirus Obsession as dependent variable.

## Data Availability

The data presented in this study are available on request from the corresponding author. The data are not publicly available due to ethical reasons.
